# LncRNA SNHG14 silencing attenuates the progression of diabetic nephropathy via the miR‐30e‐5p/SOX4 axis

**DOI:** 10.1111/1753-0407.13565

**Published:** 2024-05-16

**Authors:** YunXia Wang, JiaJia Yang, Chun Wu, Yuqin Guo, Yuan Ding, Xiujuan Zou

**Affiliations:** ^1^ Department of Renal Medicine Huai'an Rehabilitation Hospital (Jinhu People's Hospital) Huai'an China

**Keywords:** diabetic nephropathy, interstitial fibrosis, mesangial cells, SNHG14, SOX4

## Abstract

**Background:**

Diabetic nephropathy (DN) is a diabetic complication. LncRNAs are reported to participate in the pathophysiology of DN. Here, the function and mechanism of lncRNA *small nucleolar RNA host gene* *14* (*SNHG14*) in DN were explored.

**Methods:**

Streptozotocin (STZ)‐induced DN mouse models and high glucose (HG)‐treated human mesangial cells (MCs) were used to detect *SNHG14* expression. *SNHG14* silencing plasmids were applied to examine the function of *SNHG14* on proliferation and fibrosis in HG‐treated MCs. Potential targets of *SNHG14* were predicted using bioinformatics tools and verified by luciferase reporter, RNA pulldown, and northern blotting assays. The functional role of *SNHG14* in DN in vivo was detected by injection with adenoviral vector carrying sh‐*SNHG14* into DN mice. Serum creatinine, blood urea nitrogen, blood glucose, 24‐h proteinuria, relative kidney weight, and renal pathological changes were examined in DN mice.

**Results:**

*SNHG14* expression was elevated in the kidneys of DN mice and HG‐treated MCs. *SNHG14* silencing inhibited proliferation and fibrosis of HG‐stimulated MCs. *SNHG14* bound to *miR‐30e‐5p* to upregulate *SOX4* expression. In rescue assays, *SOX4* elevation diminished the effects of *SNHG14* silencing in HG‐treated MCs, and *SOX4* silencing reversed the effects of *SNHG14* overexpression. In in vivo studies, *SNHG14* downregulation significantly ameliorated renal injuries and renal interstitial fibrosis in DN mice.

**Conclusions:**

*SNHG14* silencing attenuates kidney injury in DN mice and reduces proliferation and fibrotic phenotype of HG‐stimulated MCs via the *miR‐30e‐5p*/*SOX4* axis.

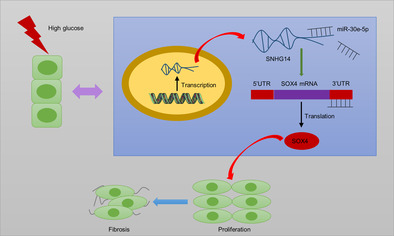

## Graphical abstract

Highlights

- *SNHG14* silencing inhibits proliferation and fibrosis in high glucose‐stimulated mesangial cells.
- *SNHG14* binds to *miR‐30e‐5p*.
- *MiR‐30e‐5p* targets *SOX4*.
- *SNHG1* *4* silencing ameliorates renal injury in diabetic nephropathy mice.

## 1 INTRODUCTION

Diabetic nephropathy (DN), a serious microvascular complication of diabetes mellitus, affects approximately 50% of patients with both type I and type II diabetes.^1^, ^2^ DN is characterized by extracellular matrix accumulation, basement membrane thickening, and glomerular hypertrophy, which are related to glomerular mesangial cell proliferation.^3^ Hyperglycemic factors including terminal glycosylation products contribute to excess extracellular matrix and lead to renal fibrosis.^4^ Glomerular mesangial cells (MCs), the main constituents of the glomerulus, are involved in the process of glomerular fibrosis by inducing the synthesis of matrix proteins.^5^ Glomerular morphological change is the main pathological damage during DN progression. Thus, preventing the proliferation of MCs is considered as a promising strategy for treatment of DN.^6^ However, the molecular mechanisms related to the alterations in the renal microenvironment are not fully understood; therefore, identifying the underlying mechanisms to discover effective biomarkers for DN or renal fibrosis is of great importance for the treatment of DN.

LncRNAs are noncoding RNA molecules with over 200 nucleotides in length.^7^ Numerous studies have reported that lncRNAs participate in the pathogenesis of human diseases.^8^, ^9^, ^10^ LncRNAs have been demonstrated to play vital roles in DN progression. For instance, upregulated lncRNA *ISG20* aggravates renal fibrosis in high glucose (HG)‐treated MCs and DN mice.^11^ LncRNA *NEAT1* modulates mitophagy to inhibit cell viability and increase reactive oxygen species production in renal tubular epithelial cells, thus accelerating damage of these cells in DN.^12^ LncRNA *MALAT* *1* downregulation protects HG‐stimulated podocytes from pyroptosis in DN development.^13^ The small nucleolar RNA host genes (SNHGs) are a special group of lncRNAs. They perform their function in the nucleus (epigenetic modulation and transcriptional regulation) and cytoplasm (translation regulation, miRNA sponging, and posttranscriptional modification), similar to other lncRNAs,^14^, ^15^ as well as influence small nucleolar RNAs (snoRNAs) at the intracellular level; in addition, they play important roles in cancer progression.^16^, ^17^ Several reports have shown the key roles of SNHG genes in the progression of DN. *SNHG1* silencing alleviates hyperglycemia and kidney injury in DN mice.^18^ Suppression of *SNHG5* protects against podocyte injury and progression of DN in mice.^19^ Silencing *SNHG15* attenuates DN progression in pediatric patients.^20^ It is noteworthy that lncRNA *SNHG14* is a newly found molecule mapping to 15q11.2 in humans^21^; it exerts diverse functions in types of human diseases.^22^, ^23^ Additionally, studies have suggested the function of *SNHG14* in renal injuries. Yang et al and Shi et al indicated that *SNHG14* was upregulated in patients with sepsis, and it promoted HK‐2 cell apoptosis and inhibited cell growth under lipopolysaccharide stimulation.^24^, ^25^ Xue et al found that silencing *SNHG14* protected against acute renal injury induced by ischemia/reperfusion in rats.^26^ These findings inspire us to investigate the function of *SNHG14* in DN‐associated renal injury. Interestingly, we found upregulation of *SNHG14* in the kidneys of DN models in this study, implying the participant of *SNHG14* in DN.

Here, streptozotocin (STZ)‐induced DN mouse models and HG‐treated human MCs were used to detect *SNHG14* expression. We evaluated the role of *SNHG14* in renal fibrosis in vitro and in vivo and investigated its underlying mechanism in DN, which may provide insight into the prevention and treatment of DN.

## 2 MATERIALS AND METHODS

### 2.1 Bioinformatic analysis

Potential miRNAs binding to *SNHG14* were predicted in starBase (https://rnasysu.com/encori/index.php) (CLIP Data: ≥12 datasets). Possible target genes of *miR‐30e‐5p* were predicted in miRDB (https://mirdb.org/), targetScan (https://www.targetscan.org/vert_72/), and starBase. The upregulated genes in the glomerulus of DN patients were shown by the dataset GSE30122 (https://www.ncbi.nlm.nih.gov/geo/query/acc.cgi?acc=GSE30122).

### 2.2 Cell culture and treatment

Human glomerular MCs were purchased from ScienCell Research Laboratories (Carlsbad, VA) and cultured in DMEM (Gibco, NY) containing 5% FBS (Gibco) at 37°C in with 5% CO_2_, followed by treatment with 5 mmol/L D‐glucose negative control (NG), 25 mmol/L mannitol, or 25 mmol/L D‐glucose (HG) for 24, 48, and 72 h.

### 2.3 Cell transfection

The shRNA targeting *SNHG14*/*SOX4* (sh‐*SNHG14*/sh‐*SOX4*) was used to silence *SNHG14*/*SOX4*. *MiR‐30e‐5p* mimics were used to overexpress *miR‐30e‐5p* with NC mimics as negative controls. Coding region of *SNHG14* or full‐length *SOX4* was planted into the pcDNA3.1 vectors to elevate the expression of *SNHG14* or *SOX4* with empty pcDNA3.1 vectors as NCs. All plasmids were purchased from GenePharma (Shanghai, China) and transfected into MCs using Lipofectamine 2000 (Invitrogen, USA).

### 2.4 Real‐time quantitative polymerase chain reaction

Total RNAs from kidneys and MCs were isolated by TRIzol reagent (Invitrogen) and were reverse transcribed to complementary DNA using reverse transcription cDNA synthesis kit (Vazyme, China), and then SYBR Premix Ex TaqTM Kit (Think‐Far Technology, Beijing, China) was used for real‐time quantitative polymerase chain reaction (RT‐qPCR) analysis on 7900HT Fast Real‐Time PCR System (Applied Biosystems). GAPDH acted as internal control. Expression of RNA was calculated with the 2^−∆∆Ct^ method.

### 2.5 Western blotting

Total proteins from MCs and homogenates of renal tissues were extracted by RIPA lysis buffer (Beyotime, Shanghai, China) and separated by 10% SDS‐PAGE followed by transfer onto PVDF membranes. The PVDF membranes were incubated with primary antibodies at 4°C overnight and then with secondary antibody at room temperature for 2 h. Primary antibodies include anti‐*p‐cadherin* (ab242026; 1:1000; abcam), anti‐*ZO‐1* (ab307799; 1:1000), anti‐*SOX4* (PA5‐72852; 1 μg/mL, ThermoFisher Scientific, USA), anti‐*fibronectin* (*FN*) (ab268020; 1:1000), anti‐*collagen IV* (*COl‐4*) (ab308360; 1:1000), anti‐*transforming growth factor beta1* (*TGF‐β1*; ab215715; 1:1000), and GAPDH (ab9485; 1:2500). Protein bands were visualized with an ECL detection kit (Bio‐Rad, Hercules, CA) and quantified with Quantity One software (Bio‐Rad).

### 2.6 EdU

EdU staining was performed according to the manufacturer's protocol of EdU Kit (Ribobio, Guangzhou, China). MCs seeded in six‐well plates at 2 × 10^5^ cells/well were fixed with 3.7% paraformaldehyde and permeabilized with PBS‐Tween‐20 for 20 min, respectively, at 25°C. Then, each well was stained with 10 μM EdU and was cultured for 2 h. After 30 min of staining with DAPI (Sigma‐Aldrich, MO), the images were observed under a Leica DM200 microscope (Leica, Solms, Germany) and analyzed with ImageJ software (National Institutes of Health, MD).

### 2.7 Immunofluorescence assay

In brief, the fixed MCs were permeabilized with 0.1% Triton X‐100. After blocking with 5% goat serum solution., the slides were added with primary antibodies against *FN* (ab268020; 1:50), *Col‐4* (ab308360; 1:500), *TGF‐β1* (ab170874; 1:50), and *SOX4* (ab243041; 2 μg/mL) at 4°C overnight and then with fluorescence‐labeled secondary antibody (Proteintech, USA, 1:50) for 1 h. After nuclei were counterstained with DAPI, the intensity was recorded using a fluorescence microscope (Olympus, Japan).

### 2.8 FISH

As per the protocols of RiboTM lncRNA fluorescence in situ hybridization (FISH) Probe Mix (Green) (Ribobio), MCs in 24‐well culture plates were fixed with 4% paraformaldehyde for 10 min, washed, and treated with 1 mL PBS containing 0.5% Triton X‐100 for 5 min at 4°C. Then 200 μL prehybridization solution was added to each well for 30 min of blocking at 37°C. Cells were mixed with 250 μL hybridization solution overnight at 37°C in the dark. Cell nuclei were stained with DAPI. Five different fields of view were captured by a laser‐scanning confocal microscope (ZEISS, Germany).

### 2.9 Luciferase reporter assay

Full‐length *SNHG14* or *SOX4* 3´UTR sequence with wildtype or mutant binding sites for *miR‐30e‐5*p was subcloned into the pmirGLO luciferase vector to construct *SNHG14*‐Wt/Mut and *SOX4* 3´UTR‐Wt/Mut vectors. The 3´UTR and coding sequences (CDS) of *SOX4* were inserted into the pmirGLO luciferase vector, denoted as Luc‐*SOX4*‐3´UTR and Luc‐*SOX4*‐CDS, respectively. All plasmids were synthesized by GenePharma and transfected into MCs using Lipofectamine 2000 for 48 h. Luciferase activity was assessed using Luciferase Reporter Assay System (Promega).

### 2.10 Biotin‐coupled probe RNA pulldown assay

A biotinylated *SNHG14* probe (Bio‐*SNHG14*) and NC probe (Bio‐NC) were synthesized by GenePharma. The probes were incubated with M280 streptavidin‐coupled Dynabeads (Invitrogen) at 25°C for 2 h to generate probe‐coated beads, which were then incubated with the cell lysates at 4°C overnight. The beads were washed with wash buffer, and the RNA complexes were then purified with TRIzol reagent and subjected to PCR analysis.

### 2.11 Northern blotting

DIG Northern Starter kit (Roche, Switzerland) was used for northern blotting analysis. Total RNA samples were denatured in formaldehyde, resolved on a 1% agarose‐formaldehyde gel, and then transferred to the nylon membranes (Beyotime). After crosslinking by ultraviolet irradiation (265 nm; 0.15 J/cm^2^), the membranes were hybridized with a biotin‐labeled DNA probe. Finally, RNA signals were measured using a Chemiluminescent Nucleic Acid Detection Module (ThermoFisher Scientific).

### 2.12 DN mouse model establishment

Male C57BL/6 mice (18–22 g and 6‐8 weeks) were obtained from Shanghai Laboratory Animal Company (Shanghai, China). Mice were maintained at 22–24°C under a 12:12 h light/dark cycle. All the protocols were conducted according to the guidelines approved by the Ethics Committee of Huai'an Rehabilitation Hospital (Huai'an, China). DN mouse models in this study were established as mentioned previously.^27^ Animals were intraperitoneally injected with 50 mg/kg STZ (S0130‐100MG‐1; LABLEAD, Beijing, China) dissolved in 0.1 M citrate buffer (pH 4.5) (Sigma Aldrich, MO, USA) daily for 5 consecutive days. The control mice received equal amounts of citrate buffer. Two weeks later, the blood glucose of mice was monitored and the animals with more than 300 mg/dL of blood glucose were considered as DN models. Four experimental groups were established: control, DN, DN + sh‐NC, and DN + sh‐*SNHG14* (*N* = 8/group). Sh‐NC or sh‐*SNHG14* (Vigene Biosciences, Shanghai) was inserted into the adenoviral vector (Life Technologies, Grand Island, NY, USA) to generate adenovirus solution of sh‐NC or sh‐*SNHG14* as previously stated.^26^ After STZ injection for 2 weeks, adenovirus solution of sh‐NC or sh‐*SNHG14* (20 μL, 10^7^ particles/μL) was delivered into the mice via the tail vein.^26^ All animals were euthanized after 8 weeks of adenovirus injection.

### 2.13 Renal function measurement

The urine of mice was collected from metabolic cages to measure 24 h proteinuria using mouse albumin ELISA kits (Abcam, USA). Animals were weighed and anesthetized with 30 mg/kg sodium pentobarbital (Sigma Aldrich) followed by cervical dislocation. The blood taken from the abdominal aorta was centrifugated at 3000 × g for 15 min to obtain the serum. An automatic biochemistry analyzer (Abbott Labs, IL, USA) was employed to measure serum creatinine and blood urea nitrogen levels. The blood taken from the tail vein was used for blood glucose measurement by a glucose analyzer (Roche). The left kidney was weighed to calculate the ratio of kidney weight to body weight.

### 2.14 Pathological evaluation of kidneys

Kidney tissues of mice were fixed in 4% paraformaldehyde and embedded in paraffin. Sections (5 μm) were cut using Rotary Microtome (Leica, Frankfurt, Germany) and stained with hematoxylin–eosin solution (H&E) (Solarbio, Beijing) and Masson's trichrome (Solarbio) as per the manufacturer's instructions. Photos were obtained by DM5000B microscope (Leica Imaging Systems). The renal tubular injury was using a scoring system as published previously.^28^ Masson‐stained areas were analyzed and quantified with ImageJ software.

### 2.15 Immunohistochemistry analysis

Renal tissue sections were treated with 0.1 M citrate buffer and boiled for 20 min in a microwave oven. After washing, samples were treated with 3% H_2_O_2_ followed by incubation in 5% BAS blocking solution for 30 min at 37°C. The rabbit anti‐*TGF‐β1* (ab215715; 1:500) and rabbit anti‐*FN* (ab268020; 1:2000) were added to incubate with sections in a wet box overnight at 4°C. After washing in PBS solution, sections were incubated with goat anti‐rabbit IgG (ab6721; 1:1000) for 1 h and stained with avidin‐biotin peroxidase complex (Solarbio) for 20 min at 37°C. Hematoxylin was used for counterstaining. The sections were photographed by an Axio Observer A1 microscope (ZEISS, Germany). Positive areas per high power field were analyzed with ImageJ software and expressed as percentage.

### 2.16 Statistical analysis

SPSS Software (Version 22.0, Chicago, IL, USA) was used to conduct statistical analyses. *p* < .05 was considered statistically significant. Statistical values are expressed as mean ± SD of five independent experiments. Student's *t* test was applied when two groups were compared. One‐way analysis of variance was performed for multigroup comparisons.

## 3 RESULTS

### 3.1 Upregulated *SNHG14* in renal tissues of DN mice and HG‐treated MCs

**FIGURE 1:**
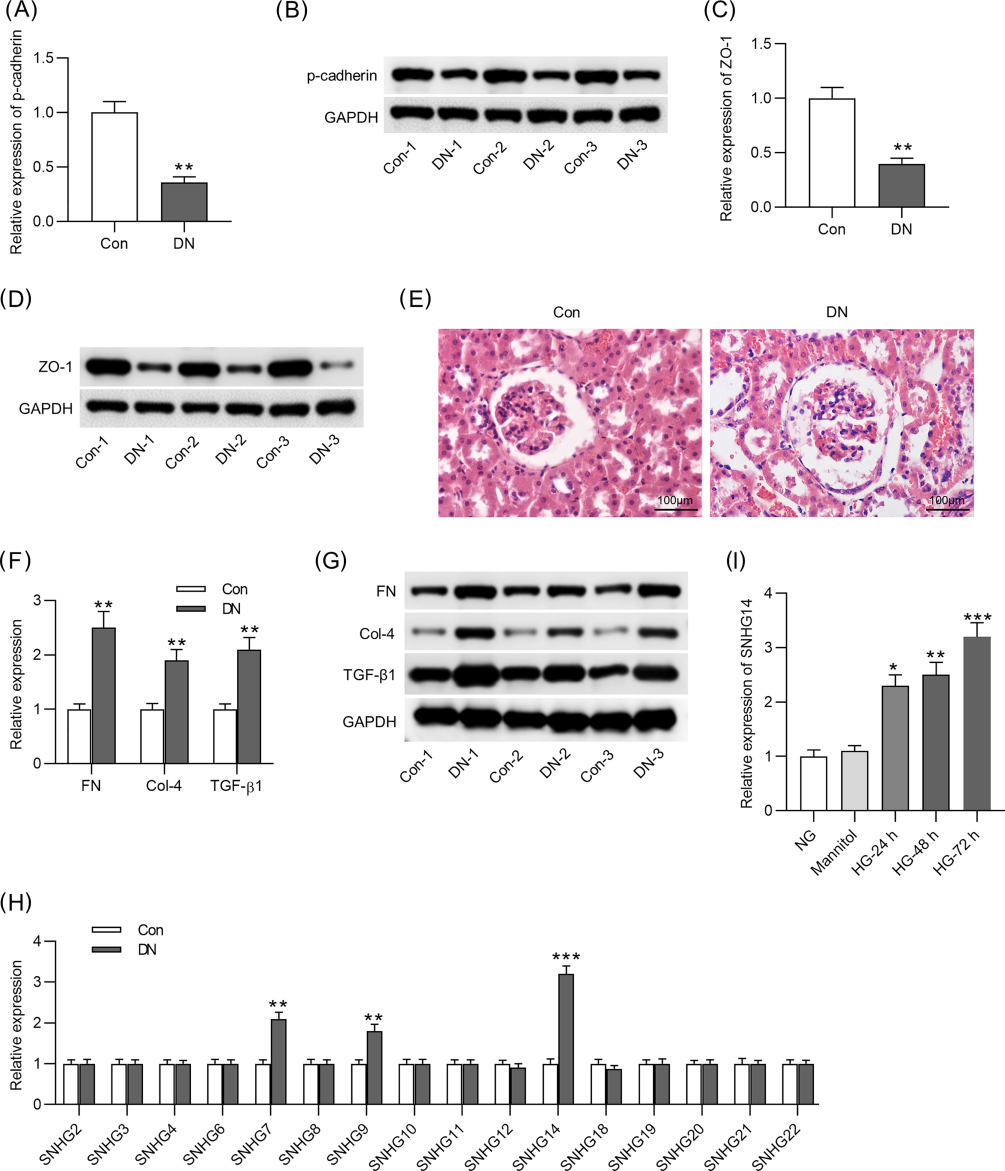
Upregulated *SNHG14* in renal tissues of DN mice and HG‐treated MCs. (A) RT‐qPCR analysis of *p‐cadherin* mRNA expression in control mice and DN mice. 0.36 fold. *N* = 3. (B) Western blotting analysis of *p‐cadheri* *n* protein expression in control mice and DN mice. *N* = 3. (C) RT‐qPCR analysis of *ZO‐* *1* mRNA expression in control mice and DN mice. 0.40 fold. *N* = 3. (D) Western blotting analysis of *ZO‐1* protein expression in control mice and DN mice. *N* = 3. (E) Renal pathological Scale bar = 100 μm. *N* = 3. (F) RT‐qPCR analysis of mRNA expression of fibrosis‐associated genes (*FN* [2.50 fold], *Col‐4* [1.90 fold], and *TGF‐β1* [2.10 fold]) in control mice and DN mice. *N* = 3. (G) Western blotting analysis of protein expression of fibrosis‐associated genes in control mice and DN mice. (H) RT‐qPCR analysis of SNHGs expression in renal tissues of mice: 2.10 fold for *SNHG* *7*; 1.80 fold *SNHG9*; 3.20 fold for *SNHG14*. *N* = 3. (I) RT‐qPCR analysis of *SNHG14* expression in MCs treated with 25 mmol/L glucose for 24 h (2.30 fold), 48 h (2.50 fold), and 72 h (3.20 fold). *N* = 3. *p < .05; **p < .01; ***p < .001. *Col‐4*, *collagen I*V; Con, control; DN, diabetic neuropathy; *FN*, *fibronectin*; H&E, hematoxylin–eosin; HG, high glucose; MC, mesenchymal cell; NG, ; RT‐qPCR, real‐time quantitative polymerase chain reaction; *SNHG14*, *small nucleolar RNA host gene 14*; *TGF‐β1*, *transforming growth factor beta1*; NG, negative control. Alt text not required.

A DN model was established by STZ injection into mouse. Compared with the control group, the levels of *p‐cadherin* and *ZO‐1* were significantly downregulated in the DN group (Figure 1A–D). Renal pathological alterations were observed by performing H&E staining. In the control mice, renal tissues showed normal glomeruli with clear tubular structure. However, dilated lumen of the renal tubules and enlarged glomeruli were detected in the DN group. There were significant interstitial inflammatory cell infiltration and vacuolarization of tubular epithelial cells (Figure 1E). Additionally, the mRNA and protein levels of fibrosis markers (*FN*, *TGF‐β1*, and *Col‐4*) were upregulated in DN mice compared with controls (Figure 1F,G). Studies have shown that there are 22 members of the SNHG family (*SNHG1* to *SNHG22*).^29^ Notably, *SNHG1*,^18^ *SNHG5*,^19^ *SNHG15*,^20^ *SNHG16*,^30^ and *SNHG17* ^31^ have been reported to participate in the development of DN. Therefore, we detected the expression levels of several SNHGs (*SNHG2*, *SNHG3*, *SNHG4*, *SNHG6*, *SNHG7*, *SNHG8*, *SNHG9*, *SNHG10*, *SNHG11*, *SNHG12*, *SNHG14*, *SNHG18*, *SNHG19*, *SNHG20*, *SNHG21*, and *SNHG22*) that have not been investigated in DN. As Figure 1H indicated, among these SNHGs, *SNHG7*, *SNHG9*, and *SNHG14* were significantly upregulated in the kidneys of DN mice compared with the control group. In addition, *SNHG14* was found upregulated in MCs by HG at 24, 48, and 72 h, compared to the control group (Figure 1I), suggesting that *SNHG14* might be related to DN pathogenesis. However, the expression levels of *SNHG7* and *SNHG9* had no significant change in the control group and HG group (data not shown). Therefore, we investigated *SNHG14* in the subsequent experiment.

### 3.2 *SNHG14* silencing inhibits proliferation and fibrosis in HG‐stimulated MCs

**FIGURE 2:**
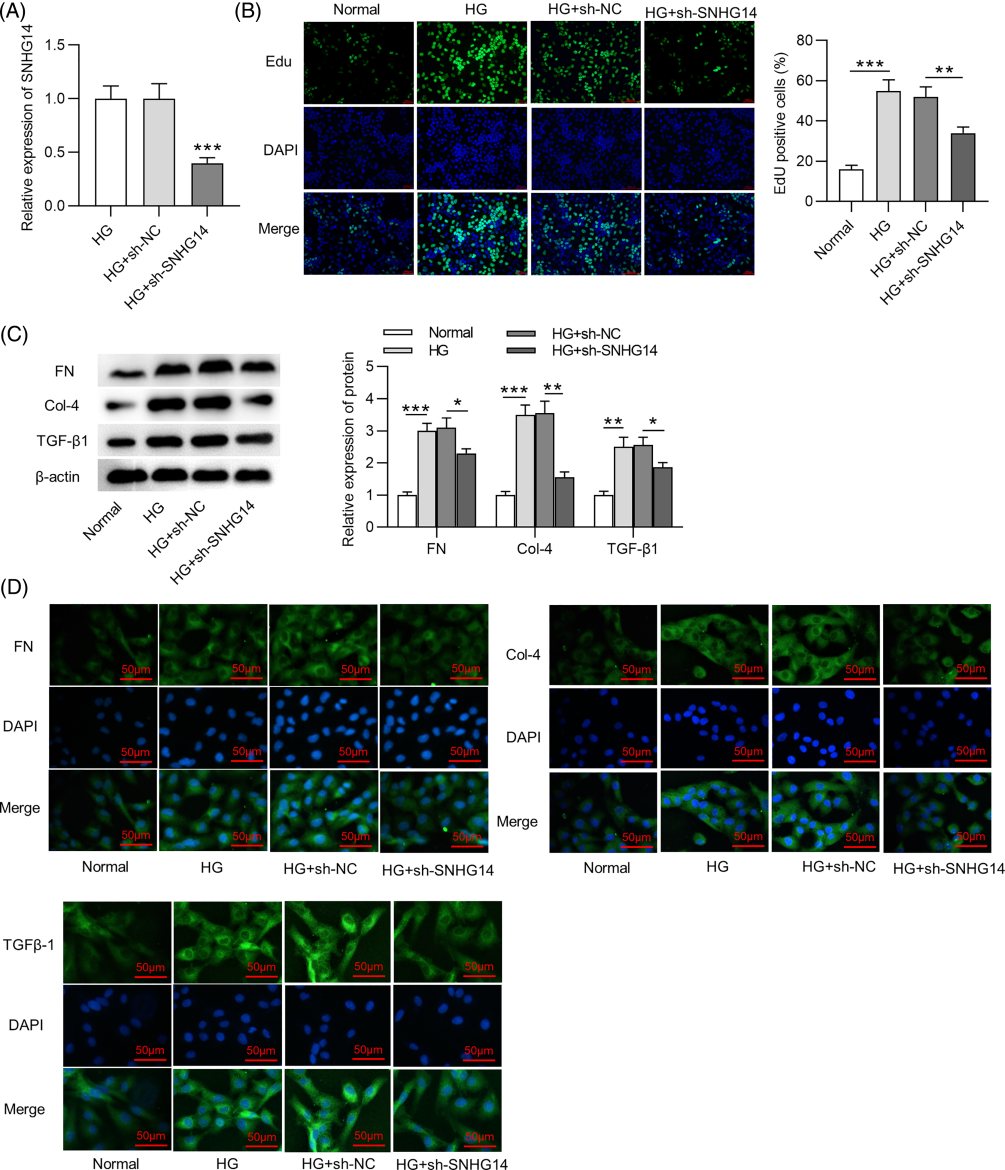
*SNHG14* silencing inhibits proliferation and fibrosis in HG‐stimulated MCs. (A) RT‐qPCR analysis of transfection efficiency of sh‐*SNHG14* in HG‐treated cells. 0.40 fold. (B) Left panel: Proliferation of MCs in different groups was detected by EdU staining. Scale bar = 100 μm. Right panel: Quantitative analysis of EdU positive cells in the normal, HG, HG + sh‐NC, and HG + sh‐*SNHG14* groups. (C) Left panel: Western blotting analysis of protein expression of fibrosis‐associated genes in different groups. Right panel: Quantitative analysis of protein expression of fibrosis‐associated genes in the normal, HG, HG + sh‐NC, and HG + sh‐*SNHG1* *4* groups. (D) Immunofluorescence staining of fibrosis‐associated proteins (*FN*, *Col‐4* and *TGF‐β1*) levels in MCs in different groups. Scale bar = 50 μm. *N* = 3. *p < .05; **p < .01; ***p < .001. *Col‐4*, *collagen IV*; *FN*, *fibronectin*; HG, high glucose; MC, mesenchymal cell; NC, negative control; RT‐qPCR, real‐time quantitative polymerase chain reaction; *SNHG14*, *small nucleolar RNA host gene* *14*; *TGF‐β1*, *transforming growth factor beta1*. Alt text not required.

Next, the role of *SNHG1* *4* in DN in vitro was explored. RNA interference (RNAi) is a natural process through which expression of a targeted gene can be knocked down with high specificity and selectivity,^32^ and it enables sequence‐specific gene silencing and can be employed to silence virtually any gene, including lncRNAs.^33^ Short hairpin RNA (shRNA) is an effective method mediating the RNAi effect.^34^ We used sh‐*SNHG14* to transfect into MCs. The RT‐qPCR analysis showed that *SNHG14* expression was reduced in HG‐treated MCs after transfection with sh‐*SNHG14* (Figure 2A). According to EdU, *SNHG1*4 silencing inhibited proliferation of MCs treated with HG (Figure 2B). In MCs with HG stimulation, *FN*, *Col‐4*, and *TGF‐β1* protein levels were decreased after silencing *SNHG1*4 (Figure 2C), suggesting that *SNHG14* downregulation represses HG‐induced fibrotic phenotype in MCs. Additionally, immunofluorescence staining yielded the same results (Figure 2D).

### 3.3 *SNHG14* binds to *miR* *‐* *30e* *‐* *5p*

**FIGURE 3:**
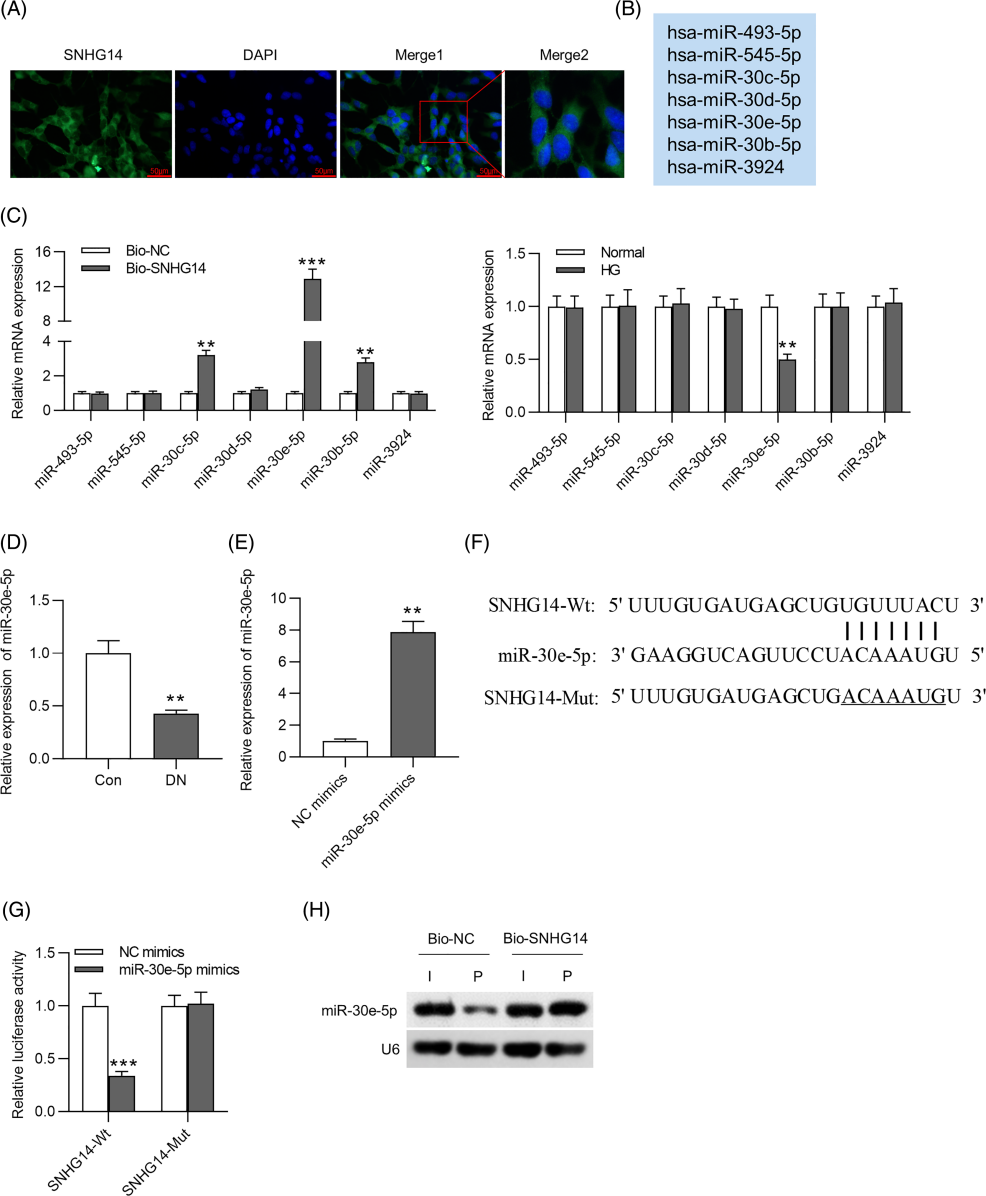
*SNHG14* binds to *miR‐30e‐5p*. (A) The subcellular localization of *SNHG14* in MCs was shown by FISH. Scale bar = 50 μm. (B) Potential miRNAs binding to *SNHG14* were predicted by starBase. (C) Left panel: RNA pulldown and RT‐qPCR analysis of miRNA expression in MCs in the Bio‐NC group and the Bio‐*SNHG1* *4* group: 3.20 fold for *miR‐30c‐5* *p*; 12.90 fold *miR‐30e‐5p*; 2.80 fold for *miR‐30b‐5p*. Right panel: RT‐qPCR analysis of miRNA expression in MCs treated with HG. 0.50 fold. (D) RT‐qPCR analysis of *miR‐30e‐5p* expression in DN mice. 0.43 fold. (E) RT‐qPCR analysis of transfection efficiency of *miR‐30e‐5p* mimics in MCs. 7.88 fold. (F) Binding sites of *miR‐30e‐5p* to *SNHG14* predicted by starBase. (G) Luciferase reporter assay of the interaction between *SNHG14* and *miR‐30e‐5p*. *N* = 3. (H) Northern blotting analysis of interaction between *SNHG1* *4* and *miR‐30e‐5p*. **p < .01; ***p < .001. Con, control; DN, diabetic neuropathy; FISH, fluorescence in situ hybridization; HG, high glucose; MC, mesenchymal cell; NC, negative control; RT‐qPCR, real‐time quantitative polymerase chain reaction; *SNHG14*, *small nucleolar RNA host gene* *14*. Alt text not required.

FISH showed that the majority of *SNHG14* was in the cytoplasm in MCs (Figure 3A), suggesting that *SNHG14* might exert functions at the posttranscriptional level. The starBase database shows thousands of miRNAs that have binding sites to *SNHG14*. We set the filter criteria as CLIP Data: ≥12 datasets and obtained seven miRNAs (*miR‐493‐3p*, *miR‐545‐5* *p*, *miR‐30c‐5* *p*, *miR‐30d‐5p*, *miR‐30e‐5p*, *miR‐30b‐5p*, and *miR‐3924*) (Figure 3B). The exact RNA sequences of *SNHG14* and potential binding miRNAs are shown in Supplementary Figure S1. To identify the miRNAs that can bind to *SNHG14*, we performed RNA pulldown assay and found that three miRNAs (*miR‐30c‐5p*, *miR‐30e‐5p*, and *miR‐30b‐5p*) showed binding capacity to *SNHG14* (Figure 3C, left panel). However, only *miR‐30e‐5p* was significantly downregulated in HG‐treated MCs among these candidates (Figure 3C, right panel). We thus selected *miR‐30e‐5p* as the research object. Additionally, *miR‐30e‐5p* was downregulated in DN mice compared with control (Figure 3D). To conduct luciferase reporter assay, we used *miR‐30e‐5p* mimics to upregulate the expression of *miR‐30e‐5p* in MCs. The overexpression efficiency of *miR‐30e‐5p* mimics was verified by RT‐qPCR. The results showed that *miR‐30e‐5p* expression was significantly upregulated after transfection (Figure 3E). Binding site of *miR‐30e‐5p* to *SNHG14* was predicted by starBase (Figure 3F). In luciferase reporter assay, *miR‐30e‐5p* mimics reduced the luciferase activity of *SNHG14*‐Wt in MCs, and neither NC mimics nor *miR‐30e‐5p* mimics affected the luciferase activity of *SNHG5*‐Mut (Figure 3G). Furthermore, the results of northern blot analysis showed that the Bio‐*SNHG14* probe could bind to *miR‐30e‐5p* in MCs (Figure 3H).

### 3.4 *MiR* *‐* *30e* *‐* *5p* targets *SOX4*

**FIGURE 4:**
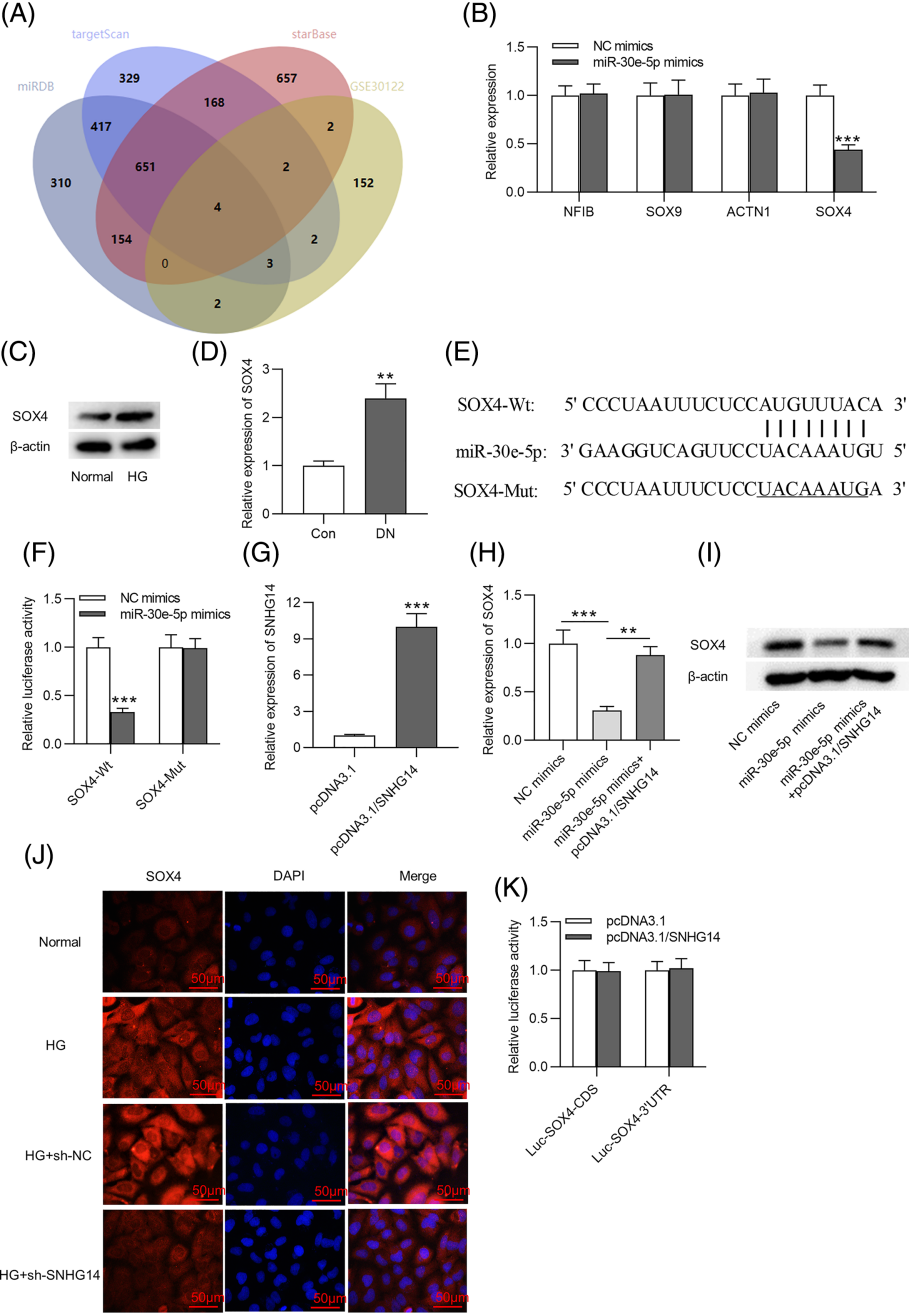
*MiR‐30e‐5p* targets *SOX4*. (A) Venn diagram showing the intersection of the targeted genes predicted by miRDB, targetScan, and starBase and the upregulated genes in the glomerulus of DN patients shown by the dataset GSE30122. (B) RT‐qPCR analysis of mRNA expression in MCs after overexpressing *miR‐30e‐5* *p*: 0.44 fold for *SOX* *4*. (C) Western blotting analysis of protein expression of *SOX4* in HG‐treated MCs. (D) RT‐qPCR analysis of *SOX4* mRNA expression in DN mice: 2.40 fold. (E) Binding sites between 3´UTR of *SOX4* to *miR‐30e‐5p* predicted by miRDB, targetScan, and starBase. (F) Luciferase reporter assay of the interaction between *SOX4* and *miR‐30e‐5p*. (G) Transfection efficiency of pcDNA3.1/*SNHG14* in MCs was detected by RT‐qPCR: 10.00 fold. (H‐I) RT‐qPCR and western blotting of the mRNA and protein expression of *SOX4* in MCs after overexpressing *miR‐30e‐5p* or *SNHG14*. (J) Immunofluorescence staining of *SOX4* expression in MCs after HG treatment or *SNHG14* silencing. Scale bar = 50 μm. (K) Luciferase activity of vector containing different region of *SOX4* was measured in cells with *SNHG1* *4* overexpression. *N* = 3. **p < .01; ***p < .001. Con, control; DN, diabetic neuropathy; HG, high glucose; MC, mesenchymal cell; NC, negative control; RT‐qPCR, real‐time quantitative polymerase chain reaction; *SNHG14*, *small nucleolar RNA host gene* *14*. Alt text not required.

To further investigate the ceRNA network involving *SNHG1* *4*, the target gene of *miR‐30e‐5p* was also explored. The miRDB, targetScan, and starBase databases are common online tools used to predict the target genes of miRNA.^35^ The GSE30122 dataset shows the differentially expressed genes in kidney tissues from patients in the DN group and normal controls.^36^ To obtain the genes that are upregulated in DN and can bind to *miR‐30e‐5p*, we used Venn diagram to screen the overlapping genes between the miRDB, targetScan, and starBase databases and the GSE30122 dataset. Four genes (*NFIB*, *SOX9*, *ACTN1*, and *SOX4*) were obtained, as shown in Figure 4A. Subsequently, we detected the expression of these four genes in MCs transfected with *miR‐30e‐5p* mimics. We found that *SOX4* was notably downregulated in MCs transfected with *miR‐30e‐5p* mimics, and the other genes (*NFIB*, *SOX9*, and *ACTN1*) had no detectable change (Figure 4B). Additionally, the protein expression of *SOX4* was elevated in MCs stimulated with HG and in DN mice (Figure 4C,D). We searched the miRDB, targetScan, and starBase databases to identify the binding site of 3´UTR *SOX4* to *miR‐30e‐5p* (Figure 4E). As Figure 4F revealed, the luciferase activity of *SOX4*‐Wt was decreased by *miR‐30e‐5p* mimics, but that of *SOX4*‐Mut was unchanged. The pcDNA3.1/*SNHG14* vector was transfected into MCs to overexpress *SNHG14* (Figure 4G). A decrease in the mRNA and protein expression of *SOX4* induced by *miR‐30e‐5p* overexpression was rescued by *SNHG14* elevation (Figure 4H,I). Immunofluorescence further demonstrated that *SNHG14* silencing reduced *SOX4* expression in HG‐treated MCs (Figure 4J). *SNHG14* has the same binding site to 3´UTR of *SOX4* (UGUUUAC) as *miR‐30e‐5* *p*. Additionally, we found the luciferase activity of Luc‐*SOX4*‐3´UTR or Luc‐*SOX4*‐CDS was unaffected in MCs with *SNHG14* (Figure 4K). This result excluded the binding of *SNHG14* to 3´UTR of *SOX4* and suggested that *SNHG14* enhances *SOX4* expression by *miR‐30e‐5* *p*.

### 3.5 *SNHG14* silencing inhibits MC proliferation and fibrosis by inhibiting *SOX4*

**FIGURE 5:**
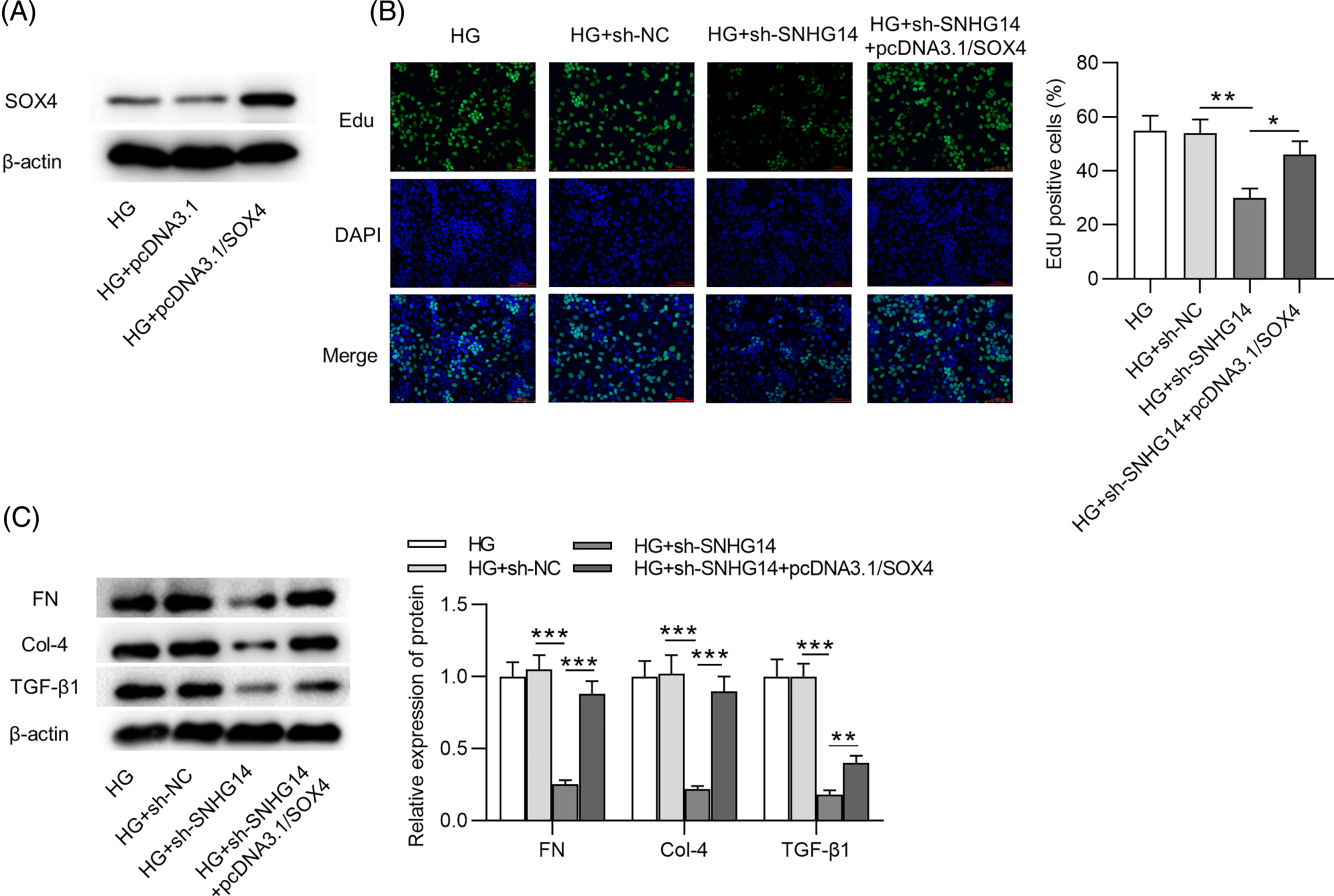
*SOX4* elevation reverses the effects of *SNHG14* silencing in HG‐treated MCs. (A) Western blotting analysis of transfection efficiency of pcDNA3.1/*SOX*4. (B) Left panel: Proliferation of MCs in different groups was detected by EdU staining. Scale bar = 100 μm. Right panel: Quantitative analysis of EdU positive cells in the HG, HG + sh‐NC, HG + sh‐*SNHG14*, and HG + sh‐*SNHG14* + pcDNA3.1/*SOX4* groups. (C) Left panel: Western blotting analysis of protein expression of fibrosis‐associated genes in different groups. Right panel: Quantitative analysis of protein expression of fibrosis‐associated genes in the HG, HG + sh‐NC, HG + sh‐*SNHG14*, and HG + sh‐*SNHG14* + pcDNA3.1/*SOX4* groups. *N* = 3. *p < .05; **p < .01; ***p < .001. FN, fibronectin; HG, high glucose; MC, mesenchymal cell; NC, negative control; *SNHG1* *4*, *small nucleolar RNA host gene* *14*. Alt text not required.

**FIGURE 6:**
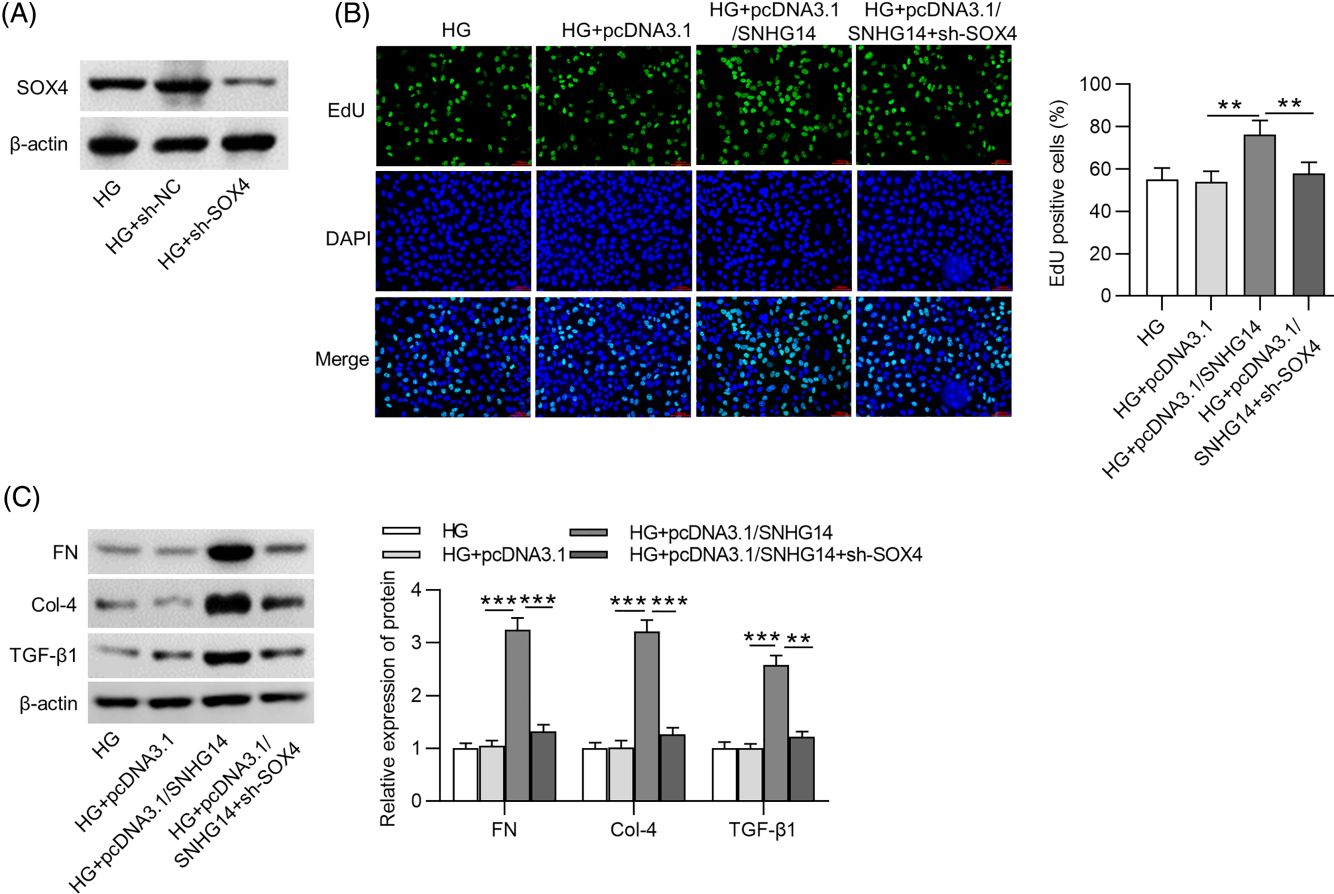
*SOX4* silencing reverses the effects of *SNHG14* overexpression in HG‐treated MCs. (A) Western blotting analysis of transfection efficiency of sh‐*SOX4*. (B) Left panel: Proliferation of MCs in different groups was detected by EdU staining. Scale bar = 100 μm. Right panel: Quantitative analysis of EdU positive cells in the HG, HG + pcDNA3.1, HG + pcDNA3.1/*SNHG14*, and HG + pcDNA3.1/*SNHG14* + sh‐*SOX4* groups. (C) Left panel: Western blotting analysis of protein expression of fibrosis‐associated genes in different groups. Right panel: Quantitative analysis of protein expression of fibrosis‐associated genes in the HG, HG + pcDNA3.1, HG + pcDNA3.1/*SNHG14*, and HG + pcDNA3.1/*SNHG14* + sh‐*SOX4* groups. *N* = 3. **p < .01; ***p < .001. *Col‐4*, *collagen IV*; HG, high glucose; NC, negative control; *SNHG14*, *small nucleolar RNA host gene* *14*; *TGF‐β1*, *transforming growth factor beta1*. Alt text not required.

In rescue assays, after transfection with pcDNA3.1/*SOX4*, the protein expression of *SOX4* in HG‐stimulated MCs was elevated (Figure 5A). EdU showed that *SOX* *4* overexpression attenuated the effects of *SNHG14* deficiency on the proliferation of HG‐treated MCs (Figure 5B). Meanwhile, after MCs were stimulated by HG, the fibrosis‐associated protein levels decreased by *SNHG14* depletion were restored by *SOX4* upregulation (Figure 5C). Furthermore, we performed rescue experiments for shRNA–mediated silencing of *SOX4*. The protein expression of *SOX4* in HG‐stimulated MCs was decreased after sh‐*SOX4* transfection (Figure 6A). EdU showed that *SNHG14* overexpression increased the proliferation of HG‐treated MCs, which was reversed by *SOX4* silencing (Figure 6B). Additionally, the fibrosis‐associated protein levels upregulated by *SNHG14* overexpression were reduced by *SOX4* silencing (Figure 6C).

### 3.6 *SNHG14* downregulation ameliorates renal injury in DN mice

**FIGURE 7:**
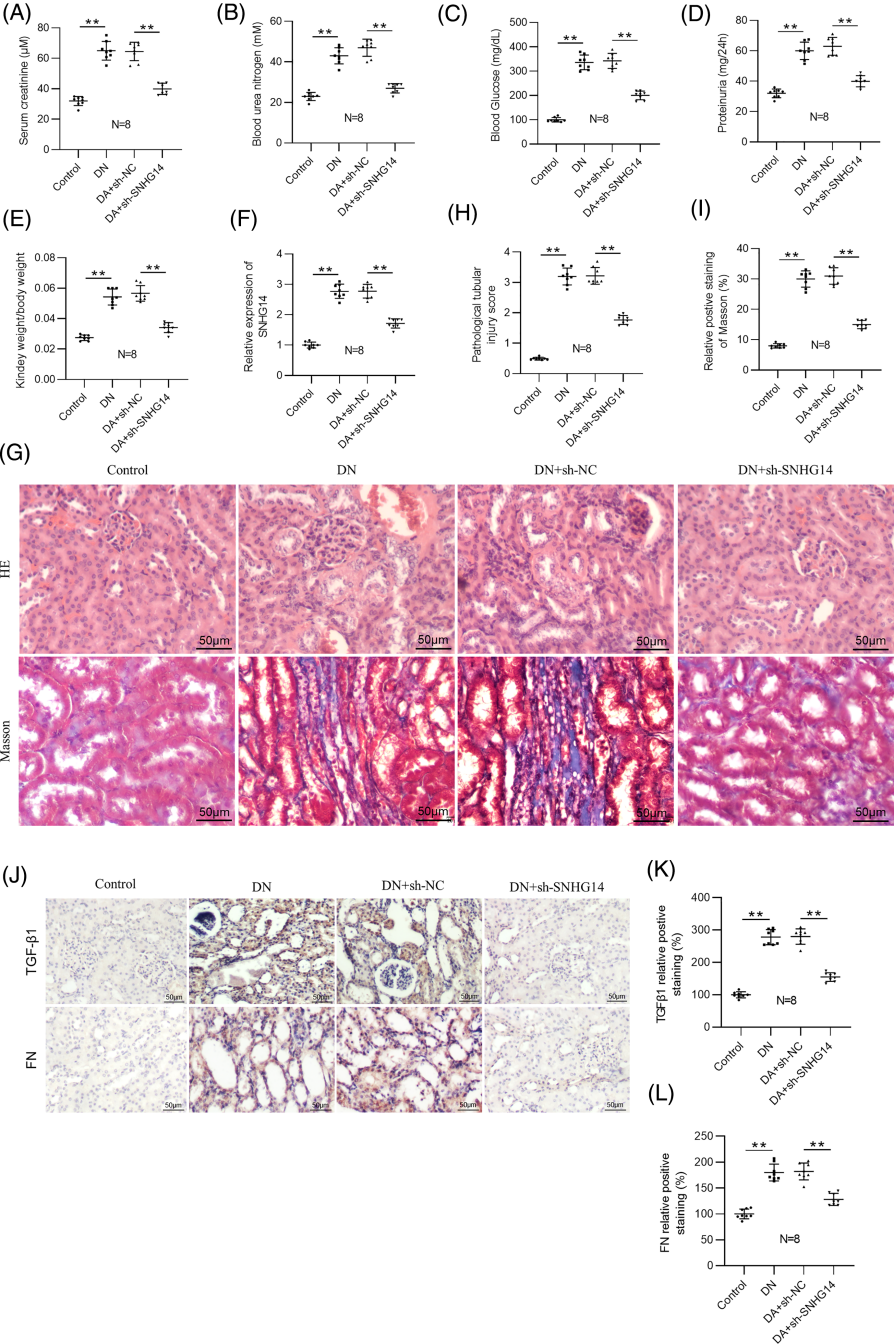
*SNHG14* downregulation ameliorates renal injury in DN mice. (A) Serum creatinine, (B) blood urea nitrogen, (C) blood glucose, (D) 24‐h proteinuria, (E) relative kidney weight, and (F) *SNHG14* expression in the control, DN, DN + sh‐NC, and DN + sh‐*SNHG14* groups. (G) H&E and Masson staining of renal tissues in the control, DN, DN + sh‐NC, and DN + sh‐*SNHG14* groups. Scale bar = 50 μm. (H) Quantification of pathological tubular injury score. (I) Quantification of Masson staining. (J–L) Immunohistochemistry analysis and quantification of *TGF‐β1* and *FN* expression in the kidneys in each group. Scale bar = 50 μm. *N* = 8. **p < .01. DN, diabetic neuropathy; *FN*, *fibronecti* *n*; H&E, hematoxylin–eosin; NC, negative control; *SNHG14*, *small nucleolar RNA host gene* *14*; *TGF‐β1*, *transforming growth factor beta1*. Alt text not required.

To investigate the function of *SNHG14* in vivo, we silenced *SNHG14* in DN mice by injection with adenoviral vector carrying sh‐*SNHG14* into DN mice. Serum creatinine, blood urea nitrogen, blood glucose, 24‐h proteinuria, and relative kidney weight were then examined in DN mice (Figure 7A–E). We found that the levels of these indicators were significantly higher in DN mice compared with control mice. However, their levels were notably reduced by *SNHG14* silencing. RT‐qPCR analysis verified upregulation of *SNHG14* in the mouse kidneys of DN, and sh‐*SNHG1* *4* induced a marked reduction in its expression (Figure 7F). H&E and Masson staining showed that sh‐*SNHG14* injection ameliorated renal pathological changes, including interstitial inflammatory cell infiltration and tubular vacuolar degeneration as well as interstitial fibrosis in DN mice (Figure 7G–I). Immunohistochemistry analysis further verified that silencing *SNHG14* downregulated *TGF‐β1* and *FN* in the renal interstitium of DN mice (Figure 7J–L).

## 4 DISCUSSION

To understand the pathogenesis of DN, an in vivo DN model was established using STZ‐administrated mice. DN mouse models exhibited an increase in DN markers (*p‐cadherin* and *ZO‐1*) and fibrosis markers (*FN*, *Col‐4*, and *TGF‐β1*) in the kidneys. Additionally, interstitial inflammatory cell infiltration and vacuolarization of tubular epithelial cells were observed in DN mice. All these pathological changes suggested a successful DN animal model. MCs are the main constituents of the glomerulus; the proliferation of MCs has been considered key contributor to renal fibrosis. Therefore, preventing the proliferation of MCs is suggested as a promising strategy for treatment of DN. Our results showed that HG stimulation promoted cell proliferative ability and upregulated fibrosis markers in MCs, suggesting that HG induced fibrotic phenotype in MCs.

Evidence has suggested the functional roles of lncRNAs in renal fibrosis prevention and DN progression. For example, lncRNA *PVT1* promotes cell migration and fibrosis of HG‐stimulated MCs to participate in DN development.^37^ *LncRNA‐NR_03351* *5* facilitates epithelial‐to‐mesenchymal transition, fibrosis, and proliferation of mouse MCs.^38^ *SNHG14* is involved in various biological processes, such as regulating neuronal cell apoptosis and inflammation,^39^ obesity‐induced endoplasmic reticulum stress in adipocyte,^40^ mesenchymal stem cell osteogenesis,^41^ cancer cell proliferation, and epithelial‐mesenchymal transition.^42^ The literature suggests the research value of *SNHG14* in human diseases. Additionally, *SNHG14* is abnormally expressed in obesity mouse models induced by high‐fat diet^40^ and in rats with renal injury.^26^ Similarly, our study showed that *SNHG14* was upregulated in DN animal models and HG‐induced human MCs. In this study, through cell experiments, we observed that *SNHG14* downregulation markedly inhibited cell proliferation and reversed fibrotic phenotype in HG‐treated MCs by decreasing *Col‐4*, *FN*, and *TGF‐β1* expression. *SNHG14* overexpression exerted an opposite result. Consistent with our findings, *SNHG14* have been reported to promote the proliferation of various cells, such as tumor cells,^43^ trophoblast cells,^44^ and atherosclerosis cells.^45^ Moreover, by adenoviral vector delivery, we demonstrated that inhibiting *SNHG14* expression also improved renal function and ameliorated interstitial fibrosis in mouse models of DN. These findings suggested a protective function for *SNHG14* silencing against DN. *SNHG1* knockdown inhibits HG‐induced ferroptosis of HK‐2 cells^18^; *SNHG5* knockdown alleviates podocyte injury^19^; silencing of *SNHG15* suppresses the inflammation in HG‐induced human glomerular mesangial cells^20^; *SNHG16* depletion inhibits the proliferation of mice mesangial cells^30^; and *SNHG17* knockdown reduces the apoptosis of podocytes.^31^ Our study was the first to show the effects of the lncRNA SNHG family on DN via the regulation of fibrosis in vitro and in vivo.

To explore the ceRNA network, potential miRNAs of *SNHG14* were predicted by online tools. The results from FISH showed that *SNHG14* was mainly localized in the cytoplasm of MCs, suggesting that *SNHG1* *4* may function at the posttranscriptional level. Numerous studies have demonstrated the involvement of miRNAs in the pathogenesis of DN through the ceRNA network.^46^, ^47^, ^48^ By luciferase reporter assay, *SNHG14* was found to bind to *miR‐30e‐5p* in MCs. Research shows that *miR‐30e‐5p* restrains hypertrophic phenotypes induced by angiotensin II in cardiomyocytes.^49^ Exosomal *miR‐30e‐5* *p* reduces HG‐stimulated pyroptosis in human renal proximal tubular cells.^50^ Moreover, *miR‐30e‐5p* expression was found reduced in the urine and plasma of patients with severe diabetic kidney disease compared with type 1 diabetes controls,^51^ and its expression was revealed to be related to the proteinuria level in DN patients.^52^ These studies have associated dysregulation of *miR‐30e‐5p* with diabetes‐related kidney disease. We also showed reduced *miR‐30e‐5* *p* expression in the kidneys of DN mice and HG‐treated MCs. We concluded that *SNHG14* may exert its function by *miR‐30e‐5p* at the posttranscriptional level. Nevertheless, the exact role and clinical relevance of *miR‐30e‐5p* in DN remain unknown and should be evaluated in the future.

To further investigate ceRNA mechanism involving in our study, the target gene of *miR‐30e‐5p* was identified. Here, we found upregulated *SOX4* in the glomerulus of DN patients from the dataset GSE30122 and demonstrated the binding of *miR‐30e‐5p* to *SOX4*. The SOX family is a critical group of transcriptional regulators implicated in various biological processes.^53^ As a well‐known transcriptional factor, *SOX4* is necessary for endocrine pancreas development.^54^ Collins et al indicated that upregulation of the diabetes gene *SOX4* suppressed insulin secretion and elevated the risk of diabetes.^55^ Additionally, *SOX4* was found to promote angiogenesis and inflammation in HG‐stimulated retinal endothelial cells, which might serve as a promising target for diabetic retinopathy.^56^ Here, our study showed that *SOX4* was upregulated in DN animal models and HG‐induced human MCs. In rescue assays, *SOX4* overexpression reversed the effects of *SNHG14* deficiency on proliferation, and fibrosis of HG‐stimulated MCs. All these findings indicated that *SOX4* was involved in the regulatory action of *SNHG14* in DN development.

There are limitations to the current work. The upstream and downstream associations between *SNHG14* and *miR‐30e‐5p* remain unknown, and the effects of the *SNHG14*/*miR‐30e‐5p*/*SOX4* axis in renal tubular compartment should be further investigated. Moreover, the mechanisms of diabetic complications are complex and are affected by environmental and genetic factors, and these need further investigations in follow‐up research.

**FIGURE 8:**
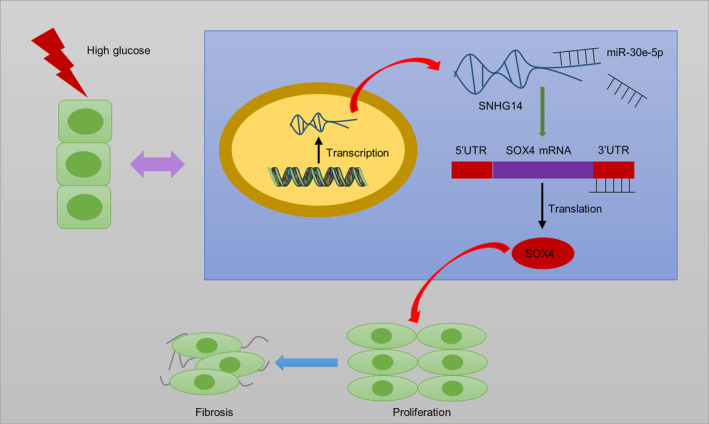
Schematic diagram showing the mechanism *SNHG14* ameliorating high glucose‐induced proliferation and fibrosis via the *miR‐30e‐p*/*SOX4* axis. *SNHG14*, s*mall nucleolar RNA host gene 14*. Alt text not required.

Collectively, *SNHG14* acts as ceRNA to elevate *SOX4* expression by sponging *miR‐30e‐5p* (Figure 8). In DN progression, *SNHG14* silencing reduces fibrosis and proliferation of HG‐stimulated MCs as well as improves renal function and ameliorates interstitial fibrosis in mouse models of DN. The present study revealed that *SNHG14* may be a potential biomarker and therapeutic target for further DN clinical application. However, a limitation in this research is that we did not pinpoint in which cell type *SNHG14* specifically exerts its function within the animal level. Further investigations need be conducted in animal models to address this issue.

## Supporting information


**Figure S1.** The exact RNA sequences of *SNHG14* and potential binding miRNAs.
