# Supplementary figures and images for: LncRNA SNHG14 silencing attenuates the progression of diabetic nephropathy via the miR‐30e‐5p/SOX4 axis
Source: J Diabetes. 2024 May 16;16(6):e13565. doi: 10.1111/1753-0407.13565 (PMC11096814; doi:10.1111/1753-0407.13565)

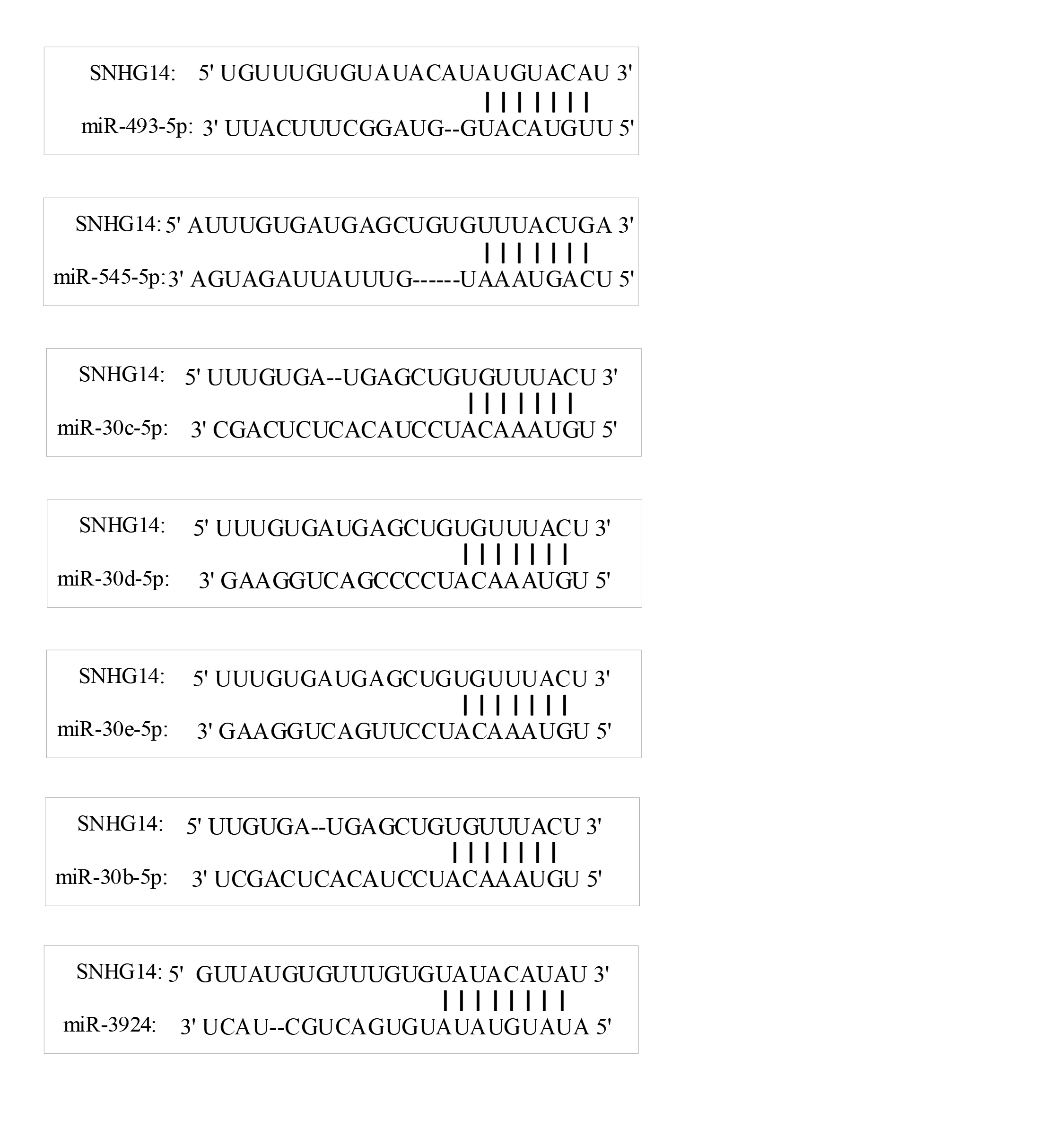

Supplement: Supplementary file 1 — Figure S1. The exact RNA sequences of SNHG14 and potential binding miRNAs. [file JDB-16-e13565-s001.tif]
